# Evaluation of a Murine Single-Blood-Injection SAH Model

**DOI:** 10.1371/journal.pone.0114946

**Published:** 2014-12-29

**Authors:** Marcel A. Kamp, Maxine Dibué, Clemens Sommer, Hans-Jakob Steiger, Toni Schneider, Daniel Hänggi

**Affiliations:** 1 Department for Neurosurgery, Medical Faculty, Heinrich-Heine-University, Düsseldorf, Moorenstraße 5, D-40225 Düsseldorf, Germany; 2 Institute for Neurophysiology, Medical Faculty, University of Cologne, Robert-Koch-Str. 39, D-50931 Köln, Germany; 3 Center of Molecular Medicine Cologne (CMMC), Robert-Koch-Str. 39, D-50931 Köln, Germany; 4 Department for Neuropathology, University Medical Center of the Johannes Gutenberg University, Langenbeckstr. 1, D-55131 Mainz, Germany; Georgia Institute of Technology, United States of America

## Abstract

The molecular pathways underlying the pathogenesis after subarachnoid haemorrhage (SAH) are poorly understood and continue to be a matter of debate. A valid murine SAH injection model is not yet available but would be the prerequisite for further transgenic studies assessing the mechanisms following SAH. Using the murine single injection model, we examined the effects of SAH on regional cerebral blood flow (rCBF) in the somatosensory (S1) and cerebellar cortex, neuro-behavioural and morphological integrity and changes in quantitative electrocorticographic and electrocardiographic parameters. Micro CT imaging verified successful blood delivery into the cisterna magna. An acute impairment of rCBF was observed immediately after injection in the SAH and after 6, 12 and 24 hours in the S1 and 6 and 12 hours after SAH in the cerebellum. Injection of blood into the foramen magnum reduced telemetric recorded total ECoG power by an average of 65%. Spectral analysis of ECoGs revealed significantly increased absolute delta power, i.e., slowing, cortical depolarisations and changes in ripples and fast ripple oscillations 12 hours and 24 hours after SAH. Therefore, murine single-blood-injection SAH model is suitable for pathophysiological and further molecular analysis following SAH.

## Introduction

Today, aneurismal subarachnoid hemorrhage (SAH) accounts for about 5% of stroke cases affecting more than 600.000 patients each year worldwide [1], [2]. Even though case-fatality has decreased over the last decades, the morbidity after aneurismal SAH remains continuously high, mostly due to insufficient treatment strategies of delayed cerebral ischemia (DCI). Despite the influence of DCI on cerebral infarction and outcome, there is increasing evidence that the mechanism of early brain injury (EBI) additionally contributes to the outcome of patients suffering from SAH. EBI is related to an early global brain injury initially after aneurysm rupture due to hypoperfusion, whereas DCI is a pathology manifesting in the subacute phase after SAH – in humans from the third day after aneurysm rupture – and an ischemia related clinical syndrome with focal neurological and cognitive deficits [3], [4]. Murine models offer several advantages compared to animal models of other species, mainly due to genetic homogeneity and the availability of transgenic animals.

Therefore, goal of the present trial was to describe the pathophysiology of EBI and DCI after experimental SAH in a murine single injection model in great detail by analyzing brain perfusion, ischemic events in the CNS, quantitative ECoG parameters and neurologic outcome, as well as cardiac disturbances.

## Material and Methods

All animal procedures were approved by the “Landesamt für Naturschutz, Umwelt und Verbraucherschutz” of the federal state of North Rhine-Westphalia, Germany (file number: 87–51.04.2010.A236).

### Animals

To study effects of SAH, 50 µl of freshly drawn blood obtained from the tail vein (or saline for the saline injection control group or no injection but perforation of the atlanto-occipital membrane for the sham group, respectively) was injected into the cisterna magna with a 30-gauge needle in order to induce SAH. Surgery was performed on 83 male C57BL/6J mice in the age range of 15–20 weeks (41 in the SAH group, 23 in the saline injection group and 19 in the sham group, each). Perfusion and cortical activity was assessed in 69 mice of which 18 were sacrificed 6 hours after injection, 11 mice 12 hours after injection, 15 mice 24 hours after injection and 25 mice 72 hours after injection. ICP was measured in 11 mice and micro CT imaging in SAH animals was performed on 3 mice.

### Animal surgery and SAH induction

General anesthesia of C57BL/6J mice was induced by intraperitoneal injection of ketamine (100 mg/kg bw i.p.), xylazine (10 mg/kg bw i.p.) and midazolam (5 mg/kg bw i.p.). Mice underwent surgery on a heating pad maintaining a consistent body temperature of 37°C. After skin incision and preparation of the skull, mice were positioned in a stereotactic frame and bilateral burr holes were placed into the scull above the S1 (−1 mm caudal and ±3 mm lateral of bregma) and the cerebellar cortex (−6.3 mm caudal and ±1 mm lateral from bregma) according to Paxinos Mouse Brain Atlas.(Paxinos and Franklin) Telemetric biopotential transmitters were implanted as previously described [5], [6]. The atlanto-occipital membrane and the foramen magnum were exposed by muscle dissection before 50 µl of freshly drawn blood obtained from the tail vein (or saline for the saline injection group or no injection but perforation of the atlanto-occipital membrane for the sham group, respectively) was injected into the cisterna magna with a 30-gauge needle in order to induce SAH over a period of 15 seconds. Mice were positioned in a head-down position for 10 minutes. The dissected neck muscles were approximated and the skin closed with sutures. Body temperature was maintained at 37°C in the postoperative recovery period. Postoperative analgesia was started after 20 minutes after intervention by subcutaneous injection of Flunixin (5 mg/kg bw s.c.). and repeated every 12 hours To prevent dehydration, additionally one milliliter saline (0.9% NaCl solution) was injected subcutaneously. Mice were kept in single polycarbonate cages under a 12 h light–dark cycle (7:00 a.m./p.m.) with food and water ad libido.

### Micro Computed Tomography

In order to verify the precision of the intra-cisternal injection, to rule out intra-cranial hemorrhage and to assess the extent of dispersion of blood in the subarachnoid space and ventricle system, micro CT imaging was performed on three mice of the SAH group. 50 µl of the CT non-ionic contrast agent Imerone 400 was added to the 50 µl blood drawn from the tail vain before injecting 50 µl of the mixture into the cisterna magna of the anesthetized mice of the SAH group. The mice were instantly placed in an ALOKA LaTheta Laboratory CT (Hitachi-Aloka, Tokyo, Japan) and scanned at 80 kV producing CT images with a pitch 0.3 mm.

### Neurologic examination

Before anesthesia and perfusion fixation, mice were blindly evaluated for neurologic deficits by one author (M.D.), using a scale previously described by Parra et al. [7] Blinded results were later assigned to the groups “SAH”, “saline injection” or “sham” by another author (M.K.). The motor score reflects evaluation of open field activity, limb symmetry, climbing and balance. The sensory score reflects evaluation of proprioception, nociception, reaction to vibrissae stimulation, eyelid reflex, olfaction and tactile response (S1 Table). A neuro-behavioral evaluation was not performed for those mice sacrificed at 6 hours post SAH, because they were still recovering from anesthesia.

### Cerebral perfusion measurement

rCBF monitoring was performed using a Laser Doppler device (moorLab, Moor Instruments, Axminster, Devon, United Kingdom) which measured relative rCBF (Flux) as well concentration (Conc) of moving blood cells. Laser Doppler probes were inserted into burr holes above the S1 and the cerebellar cortex and data collected 10 minutes before and for 30 minutes after SAH induction as well as at each of the following time points until euthanization: 6, 12, 24 and 72 hours after SAH (animals were re-anaesthetized for these later measurements). Five minutes of these recordings were evaluated and parameters expressed as relative to baseline. All Laser Doppler measurements were performed under general anesthesia. Analysis of cerebral perfusion data was performed blinded.

### Intra-cranial pressure measurement

Intra-cranial pressure (ICP) was measured in the cisterna magna using a LogiCal disposable pressure transducer neonatal monitoring kit (Smiths Medical, London, UK) connected to an M Series CCT monitoring system (Zoll Medial Cologne, Germany). The tip of a saline-filled 20 G needle, connected to the pressure transducer by saline filled tubing was inserted into the cisterna magna of the anesthetized mice. The baseline was recorded before either blood or saline injection was carried out, and ICP was measured for one minute after injection.

### Collection of telemetric data and analysis of electrocorticograms

Telemetric data from the implanted TL11M2-F20-EET transmitters were continuously collected using Dataquest A.R.T 3.1 software (Datascience International, Lexington, USA). Neuroscore 2.1.0 (Datascience International, Lexington, USA) was used to calculate absolute and relative power of frequency bands (Fast Fourier Transform based using a Hamming window). The frequency spectrum was defined as follows: Delta (0.5–4 Hz), Theta (4–8 Hz), Alpha (8–12 Hz), Sigma (12–16 Hz), Beta (16–24 Hz), Gamma (30–80 Hz), Ripples (80–200 Hz), Fast Ripples (200–500 Hz).

### Electrocardiography

Continuous electrocardiograms (ECG) could be recorded by the implanted transmitter (in addition to ECoG data) in 11 mice (7 SAH; 4 Saline) during the acute phase. Electrodes collecting the ECG signal, were implanted along with the transmitter dorsally above the left hindlimb (and not at the thoracic wall), and thus were able to record a robust ECG signal. NEUROSCORE 2.1.0 (Datascience International, Lexington, USA) was used to perform frequency domain analysis of the ECG in order to calculate LF/HF ratios immediately before (control), during and 1 minute after SAH or saline injection. FFT of the control ECG produced a periodogram with two peaks. Accordingly, for frequency domain analysis, the low frequency ECG band (LF) was defined as 0.1–1 Hz and the high frequency band (HF) as 1–5 Hz. The R-peak of the ECG QRS complex was detected automatically by Neuroscore and used to calculate heart rate and R-R intervals. The coefficient of variation (CV) was calculated from R-R intervals. Artifact-free thirty second epochs were evaluated before injection (control), immediately after injection, and 1 minute after injection.

### Fixation and perfusion

Anesthetized mice were intracardially perfused with 10% formalin followed by india ink with 10% formalin at 70 mmHg. In contrast to other authors, we did not include gelatin in the india ink-formalin mixture, because in pre-experiments we found that due to the viscosity of the solution, there was little perfusion of smaller blood vessels and high perfusion pressure was required to perfuse the larger vessels which dilated them rendering measurements of diameter pointless. To lower viscosity we also attempted to heat the solution, however the temperature required was so high i.e. supra-physiological that one must consider that infusing such a hot solution can cause severe vascular and cellular responses interfering with results. Without gelatine we were able to visualize vasculature that was not visible with the gelatin casting method and did not require high-perfusion pressure.

### Neuropathological analysis

Mouse brains with ink-stained basal vessels were photographed with a 10-fold magnification using a stereoscope (Zeiss, Oberkochenhofen, Germany). Diameter of main cerebral vessels (basilar artery, internal carotid artery, median cerebral artery and anterior cerebral artery) were determined by measuring digital images with ImageJ (http://rsbweb.nih.gov/ij/) and relating these measurements to an internal standard (0.5 mm marking) in the image. Morphological analysis was performed on brains from 10 mice (sham n = 4; saline control n = 3; SAH n = 3). Brains were first cut into parasagittal sections of 1 mm thickness using a commercially available rodent brain matrix (Rodent Brain Matrix, Adult Mouse, 30 g, sagittal Item no. RBM – 2000 S; ASI Instruments, USA) and were subsequently paraffin-embedded. Four µm-thick coronal sections were serially cut and stained with H&E and Gomori trichrome using standard protocols. For the detection of potential brain lesions injury an immunohistochemical analysis with antibodies against Iba1 (polyclonal, rabbit, no. 019-19741, WAKO) and GFAP (monoclonal, rabbit, no. 2301-1, Epitomics), respectively, was performed similarly as recently described (Frauenknecht et al., 2010). Briefly, after dewaxing, sections were incubated with a target retrieval solution (pH 9.0; DAKO, Glostrup, Denmark) at 95°C. Then, endogenous peroxidase was blocked by 3%H_2_O_2_ solution (Merck, Darmstadt, Germany) and sections were treated with FICIN (Zymed, South San Francisco, USA) at 37°C for digestion of formalin-fixed paraffin-embedded tissues. Non-specific biotin binding was blocked using a commercially available kit (Avidin/biotin blocking kit, Vector, Burlingame, CA, USA). Subsequently, sections were incubated with primary antibodies for 60 min at 21°C in a humidified chamber using the following dilutions: 1∶200 (Iba-1) and 1∶100 (GFAP). Immunoreactivity was visualized by the avidin-biotin complex method. Sections were developed in diaminobenzidine (Sigma, St. Louis, USA) with 0.03% hydrogen peroxide and counterstained with Mayers Hematoxylin. GFAP staining was performed using an immunostainer (Dako Autostainer Plus, DAKO, Glostrup, Denmark). Endogenous peroxidase was blocked by peroxidase blocking solution (DAKO, Glostrup, Denmark) Immunoreactivity was visualized by universal immuno-enzyme polymer method (Nichirei Biosciences, Tokyo, Japan). Finally, sections were developed in diaminobenzidine (Lab Vision Cooperation, Fermont, CA, USA). Omission of the primary antisera in a subset of control slides resulted in no immunostaining at all [8]–[10].

### Statistical analysis

Statistical analysis was performed using Origin 7.0 software (2012 OriginLab Corporation, Northampton, MA, U.S.A). Normal distribution of absolute ECoG power was assessed using the Shapiro-Wilk test of normality. Due to the mostly non-normal distribution absolute power values were log transformed to obtain a more Gaussian distribution [11]–[13]. and were then subjected to ANOVA, as were perfusion measurements. Coefficients of variation were below 30% and were therefore squared and subjected to the F-test for comparison of equality of variance according to Lewontin [14]. Heart-rate, LF/HF ratios and ICP values were assessed for significant differences using paired samples t-test. Data are presented as the mean ± SEM. P-values of 0.05 and below were considered significant.

## Results

### Intra-cisternal blood injection

**Figure 1:**
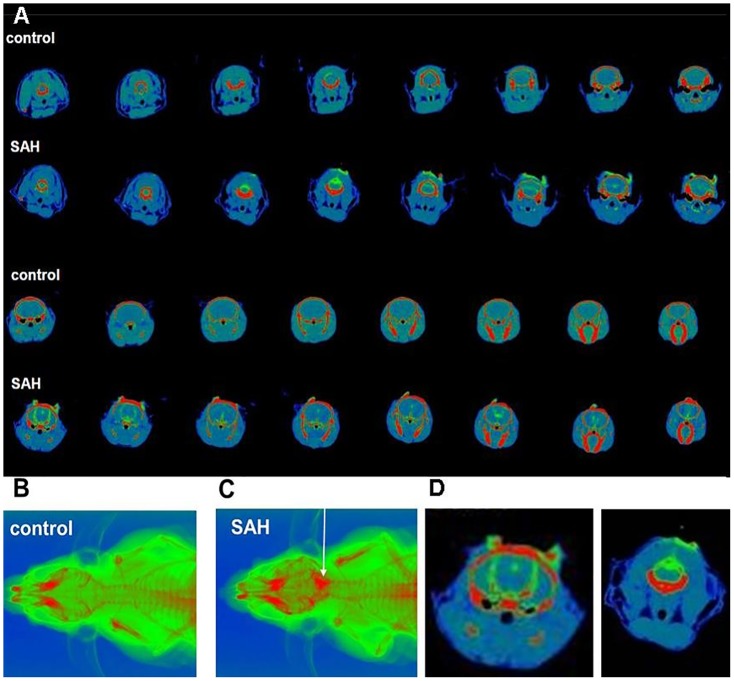
Micro CT and X-Ray imaging of cisterna magna injection. A: micro CT images (HU range −200 to 1200) collected before (control) and immediately after injection of a 1∶1 mixture of blood and the contrast agent Imerone 400 (SAH). The contrast agent is visible in the subarachnoid space and in the entire ventricular system. B: X-ray of before blood-Imerone 400 injection (control). C: X-ray collected immediately after blood-Imerone 400 injection into the cisterna magna. Note the high concentration of contrast agent in the area corresponding to the basal cistern but also quick dispersion into more cranially located regions. D: two exemplary micro images of the SAH group with a high magnification.

Micro CT imaging verified that blood (mixed with contrast agent) was successfully delivered into the cisterna magna in all scanned animals of the SAH group. Within the 20 minute scanning period, blood could be detected in all ventricles and the entire subarachnoid space (Fig. 1), confirming that cisterna magna injection of 50 µl of blood quickly results in the brain being completely engulfed by blood-containing liquor. Furthermore, accidental injection of blood into the brain parenchyma could be ruled out.

### Mortality and neurologic examination

Five of the 41 mice in the SAH group died due to the subarachnoid blood injection (mortality rate: 12.2%). Thereof, four mice died within 15 minutes after blood injection whereas one mouse died about one hour later. There were no deaths in the saline injection group and in the sham group.

Neurologic integrity (motor and sensory) was assessed 12 hours, 24 hours and 3 days after SAH (S1 Fig.). SAH impacted performance in sensory tests to a greater degree than motor performance. Animals in the SAH group displayed significant deficits in sensory tests compared to the saline group after 12 hours (SAH vs. saline 9.83±2.09 vs. 14.42±0.3; p = 0.014 U = 4) and compared to saline and sham groups after 24 hours (SAH vs. saline: 11.16±0.79 vs. 14.71±0.81; p = 0.0012 U = 0; SAH vs. sham: 9.83±2.09 vs. 10.5±1.19; p = 0.038 U = 2.5). After 3 days, although differences in sensory or motor performance of SAH animals compared to both control groups were not significant, comparing the sum of sensory and motor scores (to a total neurologic integrity score) revealed significant differences, indicating persistent delayed effects of SAH. After 3 days, total neurologic integrity in SAH animals was 16.2% lower compared to saline-injected animals (p = 0.0317 U = 1.5) and 18.52% lower compared to sham-operated animals (p = 0.015 U = 2.0).

### Cerebral perfusion and ICP following intracisternal blood injection

**Figure 2:**
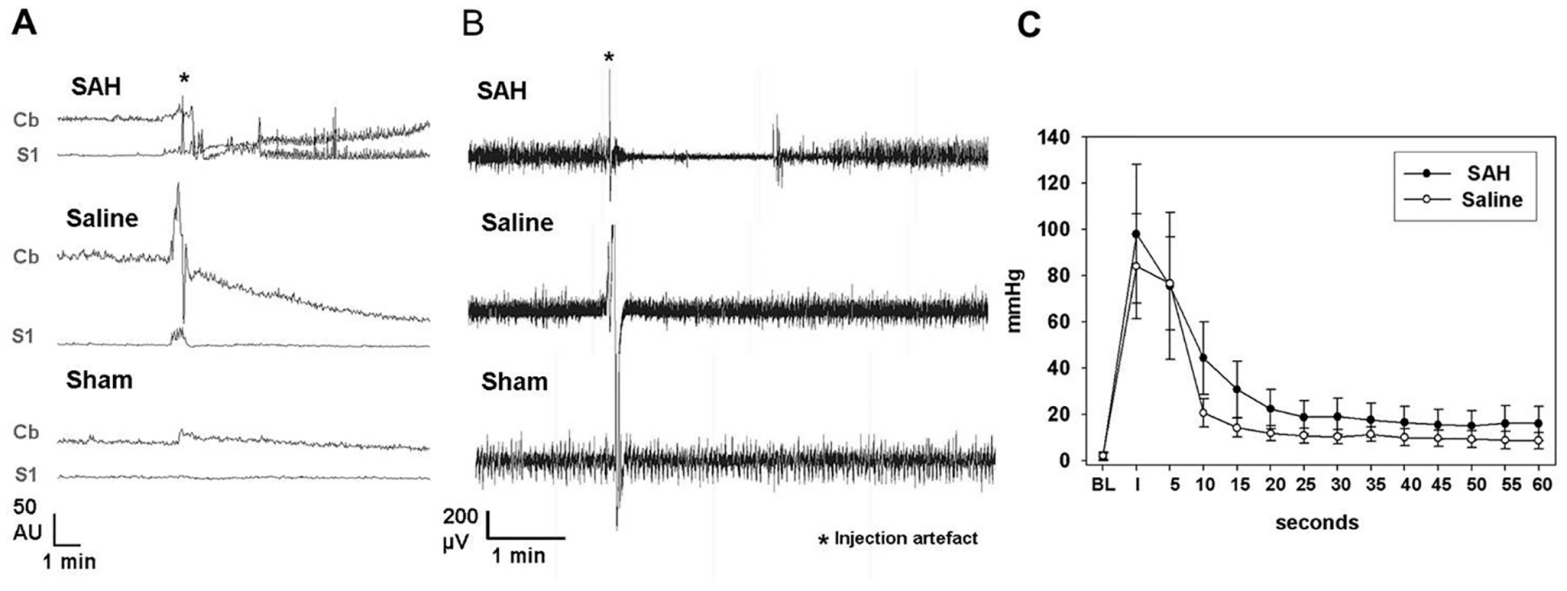
Acute effects on perfusion, cortical signaling and ICP. A: sample traces of rCBF of the three experimental groups immediately after injection. B: sample ECoG traces of the three experimental groups immediately after injection. C: ICP in SAH and saline groups before, during and until 1 minute after injection. Curves of the two groups do not significantly differ from each other. Both groups display significant elevation from baseline in the first 20 seconds after injection in paired samples t-test. Interestingly, although no longer statistically significant, in both groups, ICP settles at a new plateau that is higher than baseline for the rest of the recording period.

An acute impairment of regional cerebral perfusion (rCBF) and concentration of the moving blood cells (Conc) measured by Laser Doppler Flowmetry above the somatosensory cortex (S1) and the cerebellar cortex was observed immediately after injection in the SAH group and in about half of animals of the saline injection group but not in sham operated animals (Fig. 2, S2 Fig.). Typically, cerebral perfusion decreased till a nadir was reached, recovered in the surviving animals of the SAH reaching a peak and then a new plateau that frequently was lower than the initial baseline (S2 Fig.).

Quantification of maximal acute cerebral hypoperfusion following injection measured at the rCBF nadir revealed significant differences of the absolute values of cerebral and cerebellar rCBF and cerebellar Conc between the SAH and saline injection group (mean cerebral rCBF: 9.32±1.8 vs. 26.4±7.0; p = 0.002; mean cerebellar rCBF: 25.6±4.9 vs. 61.3±13.8, p = 0.006; mean cerebellar Conc: 83.2±10.6 vs. 168.5±17.3, p = 0.00012). As perforation of the atlanto-occipital membrane in the sham group did not lead to changes in cerebral and cerebellar perfusion, nadir values of the SAH and saline-injection group were compared to the values immediately after perforation of the atlanto-occipital membrane: Comparison of these values revealed significant differences between the sham group and the SAH or the saline-injection group in cerebral rCBF (mean cerebral rCBF for the sham group: 29.7±3.7; p = 0.004 and p = 0.015, respectively) and between the sham operated and SAH group for the cerebellar rCBF (mean cerebellar rCBF for the sham group: 149.3±20.1; p = 0.003).

Relative perfusion values may give a better insight in the mechanisms following SAH (S2 Fig.; S2 Table): For cerebral and cerebellar rCBF, relation between the nadir and the baseline as defined before injection was significantly lower in the SAH than in the saline-injection group as well as to the early baseline after perforation of the atlanto-occipital membrane (S2 Fig.). Furthermore, the relation between the nadir and the following peak significantly differed between the SAH and sham operated group. As the sham operated group exhibited no perfusion impairment, these values were not defined. Time difference between nadir and following peak was not significantly different (SAH group: 289 s±144 s vs. saline injection group: 28 s±16 s, p = 0.18).

Upon injection, ICP immediately surged from baseline (SAH: 1.57±1.67; saline 2±1.64) to similar values in both SAH and saline groups (SAH: 98±30; 84±22.8). Both groups display statistically significant elevation of ICP in the first 20 seconds after injection compared to baseline in paired-sample t-test (Fig. 2C). Twenty seconds after injection, ICP had recovered from extreme elevation, however continued to be significantly elevated in both groups (SAH 18.7±7.2 vs. 1.57±1.67 p = 0.046; saline 10.7±3.2 vs. 2±1.64 p = 0.002). Although no longer significant after 30 seconds, in both groups ICP values continued to be elevated compared to baseline throughout the remaining recording period. ICP curves did not significantly differ between SAH and saline groups.

### Cerebral perfusion measurement of delayed ischemia

**Figure 3:**
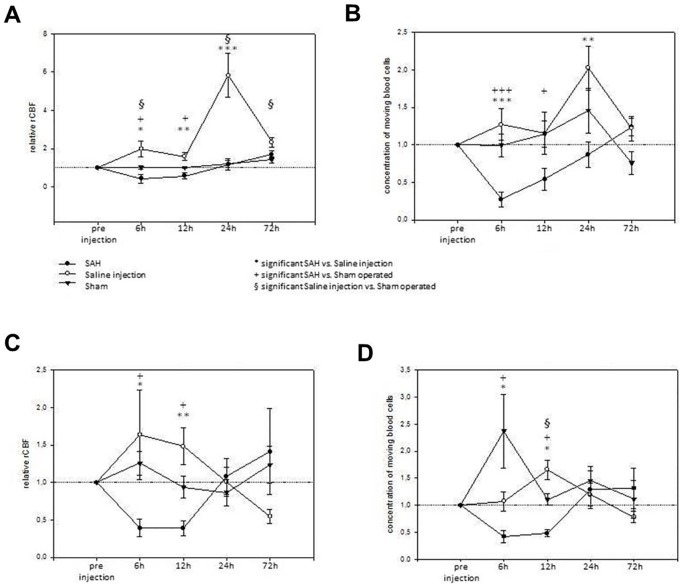
Cerebral and cerebellar rCBF impairment during DCI. Fig. 3 summarizes impairment of regional cerebral blood flow and the concentration of moving blood cells in the S1 and cerebellar cortex related to pre-injection values at time points 6, 12, 24 and 72 hours after injection. A: relative rCBF in the S1 cortex. B. concentration of moving blood cells in the S1. C. relative rCBF in the cerebellar cortex. D. concentration of moving blood cells in the cerebellar cortex. Note the delayed perfusion impairment in the SAH group 6 and 12 hours after injection and interestingly, the significant and long-lasting hyperperfusion that is caused by saline injection.

To determine intra-individual perfusion changes after SAH, the relative change from baseline was determined for Laser Doppler parameters. In S1, injection of saline induced a robust and long-lasting increase in rCBF (maximum: 580% from baseline after 24 hours) and Conc (maximum: +202% from baseline after 24 hours) lasting from 6 to 24 hours after saline injection, whereas SAH decreased rCBF (minimum: −43% from baseline after 6 hours) for 12 hours and Conc (minimum: −27% from baseline after 6 hours) for 24 hours after SAH (Fig. 3). Sham operated animals exhibited no marked changes of their cerebral and cerebellar rCBF from the baseline (maximum: 25% from the baseline after 6 hours) but a marked increase of Conc within the cerebellum after 6 hours (236% from the baseline). Decrease of S1 rCBF in SAH animals reached significance compared to the saline injected animals after 6, 12 and 24 hours and compared to the sham operated animals after 6 and 12 hours after injection (S3 Table, Fig. 3). Also cerebellar rCBF and Conc was significantly reduced compared to both control groups after 6 and 12 hours. In all groups, near-to baseline levels of both rCBF and Conc had been reestablished after 3 days. The effects of SAH on rCBF and Conc in the cerebellar cortex are similar to those measured in the S1, however baseline conditions are reestablished earlier at 24 hours after SAH. Interestingly, in the sham group hyperperfusion is also measured in the cerebellar cortex but much less pronounced than in the S1 (116% vs. 296% at 24 hours after saline injection; p = 0.006).

### Electrocorticography

**Figure 4:**
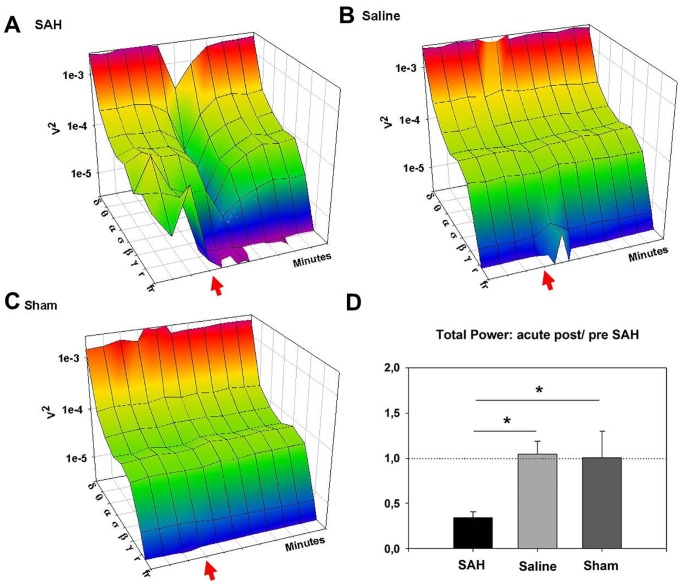
ECoG spectrum following SAH, saline and sham injection. Fig. 4 depicts the effect of the three experimental conditions on the ECoG spectrum. A: power spectrum of the SAH group. Injection of blood causes a drastic reduction of absolute power of almost all frequency bands lasting several minutes. Fast ripples were not detectable before SAH, but emerge instantly thereafter. B: power spectrum of the saline-injected group. C: power spectrum of the sham-injection group. Fast ripples were not detectable in saline or sham injected groups. D: Total ECoG power was significantly reduced in the SAH group by an average of 65%.

**Figure 5:**
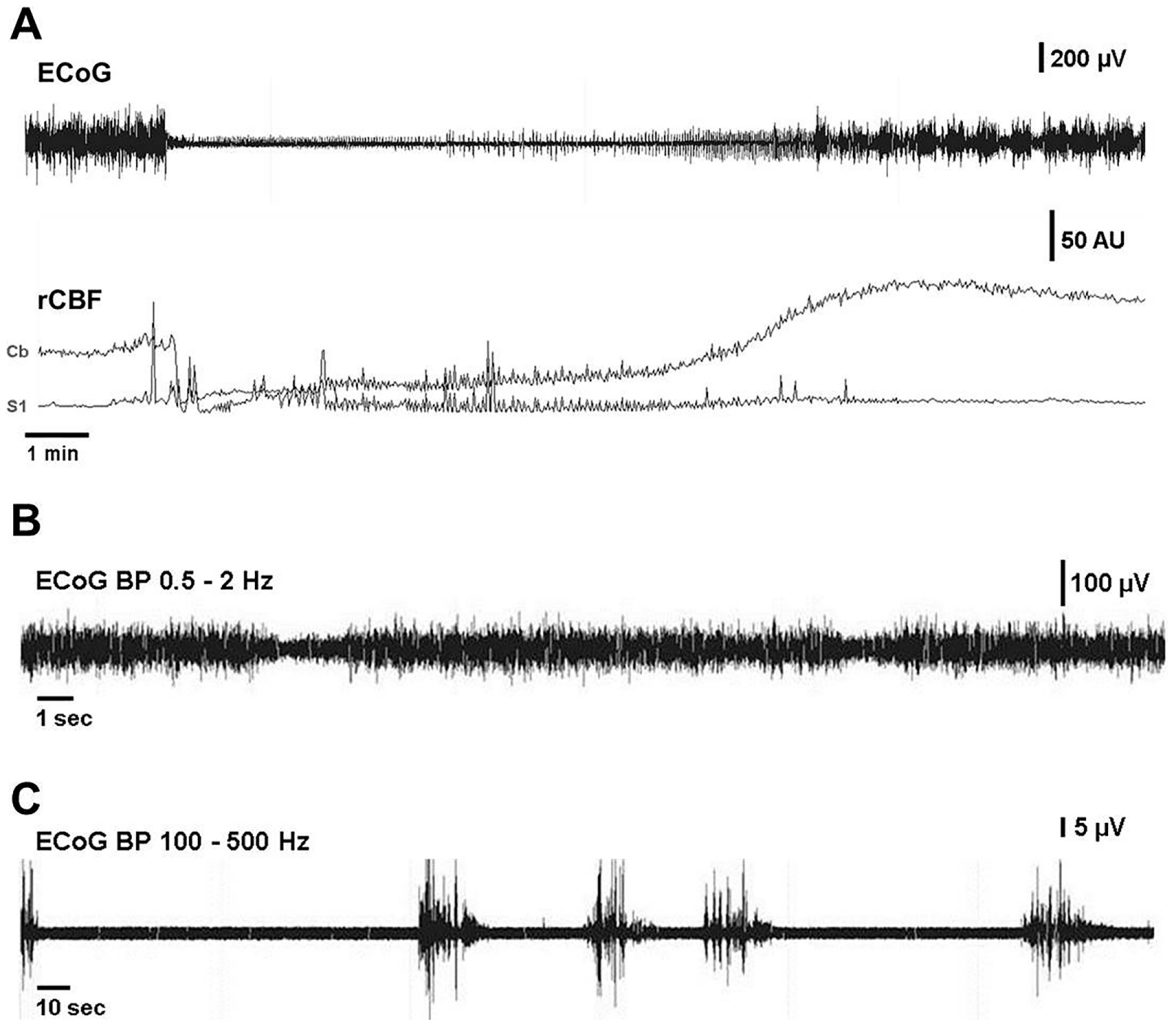
Exemplary ECoGs during acute SAH. A: Induction of SAH causing immediate severe perfusion impairment corresponds to complete cessation of ECoG signal in some animals lasting several minutes with activity returning in burst suppression patterns. Interburst intervals became continuously shorter until pre-injection levels of total power were reached. B: Bandpass (0.5–2 Hz) filtered ECoG. Recurrent short episodes of depression, potentially corresponding to cortical spreading depression were recorded in the low-frequency range in SAH (n = 3) but not in either control group. C: Bandpass (100–500 Hz) filtered ECoG. In SAH animals, short bursts in the ultra-high frequency spectrum occurred about 24 hours after SAH.

Telemetric electrocorticograms and electrocardiograms were continuously recorded before induction of SAH and until animals were sacrificed. Spectral analysis of electrocorticograms (ECoGs) was performed to evaluate specific differences in cortical activity among the different animal groups. There were no differences in spectral distribution between the 3 groups before injection. Injection of blood into the foramen magnum instantly reduced total ECoG power by an average of 65% (from 2.6 mV±0.7 mV to 0.9 mV±0.3 mV), whereas injection of saline (or sham injection) did not reduce total ECoG power at all (SAH vs. Saline p = 0.00016; SAH vs. Sham p = 0.02; Fig. 4). The reduction of total ECoG power in the SAH group compared to both control groups reflects significant reduction of absolute power in all frequency bands except the two high frequency bands (ripples and fast ripples; Fig. 4 A–C). This robust depression of cortical activity (complete cessation of ECoG signal in some animals) lasted for several minutes with activity returning in burst suppression patterns (Fig. 5A). Interburst intervals became continuously shorter until pre-injection levels of total power were reached. Furthermore, although it did not reach statistical significance, it may be important to note that absolute power of fast ripple oscillations appeared to increase in the first few minutes after intracisternal injection of blood, but not in the two control groups. Four of the five animals that died after subarachnoid blood injection, exhibited complete cessation of the ECoG signal with a parallel decrease of cerebral perfusion. In those four animals, both cerebral perfusion and ECoG activity did not return so that the animals died within a few minutes after subarachnoid blood injection. One animal died about 2 hours after subarachnoid blood injection.

SAH animals displayed significantly increased absolute delta power i.e. slowing 12 hours and 24 hours after SAH (p = 0.022 and p = 0.041 respectively), which is also reflected by a reduction in alpha/delta ratio (ADR) of SAH animals compared to saline-injected animals 24 hours after SAH (0.37±0.09 vs. 0.64±0.06; p = 0.038). Periodically recurring short episodes of depression of activity within the 0.5–2 Hz range lasting 2–3 seconds were observed in some SAH animals but not in saline-injected or sham-operated animals (Fig. 5B), likely reflecting spreading depolarization, which are observed as abrupt, large, negative slow potential changes measured in the low-frequency or direct current (DC) range of the ECoG [15]. After 3 days, no differences in ECoG spectra were visible between the three groups. In SAH animals, bursts in the high-frequency spectrum occurred about 24 hours after SAH (Fig. 5C).

### Electrocardiography

**Figure 6:**
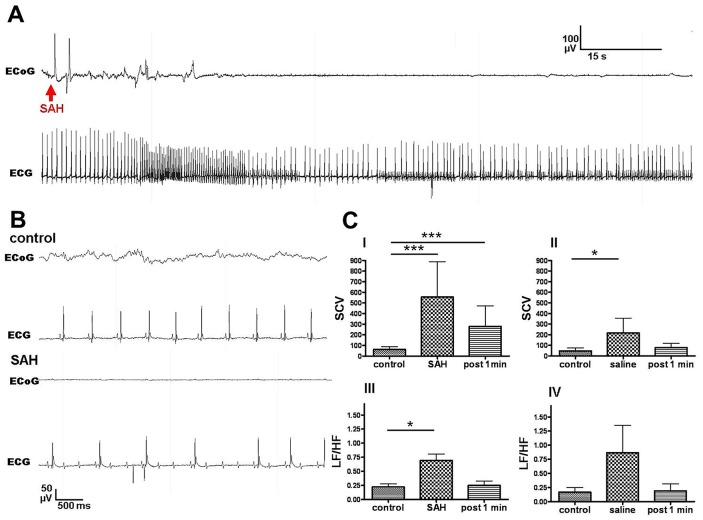
Cardiac responses to SAH induction. Fig. 6 depicts a sample ECG trace after SAH. Three out of seven mice in the SAH group displayed transient high grade second and/or third degree atrioventricular block during flat-lining of the ECoG shortly after SAH (Figs. A and B bottom). F-test for comparison of equality of variances revealed increased SCV in SAH mice (C I) and in saline-injected mice (C II). SAH mice also display a transient significant increase in LF/HF ratio, potentially reflecting sympathetic activation to stabilize CPP after SAH or a Cushing reflex due to elevated ICP (C III).

Continuous telemetric electrocardiograms (ECG) could be recorded in 11 mice (7 SAH; 4 Saline) during the acute phase. During the control condition heart rate and squared coefficient of variation (SCV) did not differ between SAH and saline groups (heart rate: 145±10 bpm vs. 160±36 bpm; SCV: 69.7±25 vs. 47.4±29.4). One minute after blood injection, heart rate dropped from 145±10 bpm to 113±11 bpm in the SAH group, however this reduction did not reach significance (p = 0.07). Heart rate was not affected by saline injection (159±36 bpm vs. 151±21 bpm). The SCV was significantly increased immediately after blood (p<0.0001) and saline (p = 0.027) injection compared to the control condition (Fig. 6C I and II). The LF/HF ratio, considered to be a measurement of sympatho-vagal balance, was significantly increased in the SAH group immediately after injection (Fig. 6C III), however the similarly robust increase in LF/HF in the saline group did not reach significance in paired-samples t-test due to high inter-individual variability and lower sample size in this group (n = 4). In three out of the seven SAH mice, flat-lining of the ECoG immediately after blood injection coincided with high-grade second degree and/or third degree atrioventricular block (AV, Fig. 6 A and B). In the saline group only one out of four mice displayed a first degree AV block.

### Neuropathological analysis

Analysis of vessel diameter of basal cerebral arteries in ink-perfused whole brain preparations revealed no differences between the SAH and the control groups at 6, and 24 hours (S3 Fig.). Although there was a trend for a reduction in the diameter of MCA and PCA of SAH animals at 24 hours post injection compared to 6 hours post injections, this did not reach statistical significance (MCA 57.2±12.3 vs. 113.4±20.3; PCA 98±17.7 vs. 127.5±19.6). In a subset of animals of all experimental groups sacrificed at 3 days after SAH (n = 10), neuropathological analysis revealed trough-shaped lesions corresponding to the location of the inserted electrodes or Laser Doppler probes (each 3 mice in the sham and SAH group, respectively). Apart from these lesions, examination of HE, GFAP and lba1 stained sections did not reveal any obvious signs of infarction, gliosis or inflammation in any of the three experimental groups.

## Discussion

The murine single-blood-injection SAH model elicits early impairment of cerebral perfusion and cortical signaling and delayed neurologic deficits and ECoG slowing. SAH models are well-documented in other rodents than mice such as rats [16]–[22]. A murine SAH injection model which analyses and correlates cerebral perfusion, neurologic function, cortical signaling, intracranial pressure and standard histopathological results is yet not available but would be a prerequisite for further transgenic studies assessing the mechanisms following SAH. Therefore, a murine SAH injection model allowing systematic assessment of early brain injury as well as delayed cerebral vasospasm after SAH poses several advantages due to the great knowledge about mouse physiology and availability of technical resources and transgenic animals.

Our present model analyses two different aspects of the pathophysiological changes after SAH which are the EBI with an early global brain injury initially after aneurysm rupture and DCI as a pathology manifesting in the subacute phase after SAH as an ischemia related clinical syndrome with focal neurological and cognitive deficits. DCI was evaluated for the first time in a murine SAH filament perforation model evaluating the constriction of in the late 1990′s [23]. A more detailed analysis of the murine SAH filament perforation model was performed a few years later [7]. In this model, the ICA and the distal MCA is constricted compared to sham controls at 72 hours after SAH correlating to impaired neurological function and SAH grade [7], [23]. Besides the main cerebral vessels, arterioles are frequently constricted and occluded by microthrombi up to 3 days after induction of SAH in this endovascular model in vivo [24]. Later, a murine blood injection model was introduced, which involved injecting blood through the atlanto-occipital membrane into the cisterna magna and analyzing cerebral vessel diameter after injection [25]. The investigators suggested an initial phase of vasoconstriction peaking around 6 to 12 hours after SAH induction and a second phase of vasoconstriction between day 1.5 and 4. However, the MCA was significantly constricted only between 6 and 36 hours [25]. In a prechiasmatic SAH model, a needle was stereotactically introduced through the brain to inject blood into the prechiasmatic cistern [26]. Initially, rCBF decreased after injection in both SAH and the saline-injection groups and recovered to a lower baseline after about 10 minutes [26]. In the SAH group, MCA and ACA were significantly constricted after 7 days and analysis of brains revealed brain injury with induction of apoptosis and neuronal degeneration [26]. Also for the prechiasmatic cistern SAH injection model, arterioles constriction and microthrombi were shown [27]. Several modifications of murine SAH models assessing DCI have been described, a correlation between cerebral perfusion, vessel diameter, cortical signaling, neurologic integrity and histopathological changes in one model has yet not been performed.

Initial responses and EBI after SAH has been modeled in a murine filament perforation model in which the ICP increased sharply and the rCBF drops by about 80% in response to SAH [28]. In addition to other limitations, the filament perforation model does not allow differentiation between specific effects of subarachnoid blood and mass effects evoked by the additional subarachnoid volume and increase of ICP. By using suitable controls, such as a control saline injection, effects of subarachnoid blood can be differentiated from other effects in a blood injection model. EBI in a murine blood injection model has only been analyzed in a further single study, in which blood was injected into the prechiasmatic cistern by stereotactically introducing a needle through the brain [26]. Initially, rCBF decreased after injection in both the SAH and the saline-injection groups and recovered to a lower baseline after about 10 minutes. No differences in rCBF decrease were described between the SAH and saline injection group. However, although both studies addressed EBI in murine SAH models, a comprehensive analysis of EBI in a murine model had yet to assess the correlation between cerebral perfusion impairment and neuronal signaling and specific effects thereof.

The present study aimed to analyze impairment of cerebral perfusion and neuronal signaling measured by ECoG following SAH in a murine blood injection model. Mortality rate was 12.2% in the SAH group but there were no deaths in the saline injection and the sham group. ICP curves of both saline and blood injected animals were similar, displaying a robust surge upon injection lasting 20 seconds corresponding to the acute impairment of regional cerebral perfusion observed immediately after injection in the SAH group and in most animals of the saline injection group but not in sham operated animals. Quantification of acute absolute and relative hypoperfusion following injection revealed significant differences of cerebral and cerebellar rCBF and cerebellar Conc between the SAH and saline injection groups. The degree of cerebral hypoperfusion significantly differed between the SAH and both control groups. An acute hypoperfusion after SAH is in line with various previous studies. In part, increasing volume in the subarachnoid space or shifting of the subarachnoid milieu appears to impair cerebral perfusion as it also dropped in some animals of the saline-injection group. However, absolute and relative cerebral perfusion values were significantly lower after SAH compared to the saline-injection indicating a specific role of subarachnoid blood in the genesis of cerebral hypoperfusion. Furthermore, injection of blood into the foramen magnum reduced total ECoG power by an average of 65%, whereas injection of saline (or sham injection) did not reduce total ECoG power. Although it did not reach significance, fast ripples, pathological high frequency oscillations known to precede epileptic seizures, were barely detectable prior to SAH but became robustly detectable in several mice after blood but not after saline injection. To our knowledge, the present study is the most comprehensive analysis of acute ECoG changes following SAH and the only one using quantitative ECoG methods in an animal model. Previously, suppression of EEG delta activity in the electrical cortical activity has been described in a rat animal model immediately after SAH which is supported by our findings [16]. We presently did not focus on a systematic analysis of electrocardiographic activity after SAH, however acute cardiac responses could be observed in SAH and saline groups. The increase of the SCV in both groups upon injection may reflect a transient arhythmogenic affect of increased ICP. Interestingly, severe AV block was only observed in SAH animals (3/7). Furthermore a transient increase in the LF/HF ratio may be indicative of reactive sympathetic activation in order to increase blood pressure and thereby CPP or related to a Cushing reflex due to transient ICP elevation.

Furthermore, the present study analyses delayed cerebral ischemia after SAH in great detail. Significant impairment of rCBF in the S1 occurred between 6 and 12 hours after induction of SAH in comparison to both sham-operated and saline injected animals which was accompanied by a reduction of the concentration of moving blood cells at 6 and 12 hours. In the SAH group, rCBF and Conc were within the pre-injection range after 24 hours, whereas saline-injected mice exhibited significant hyperperfusion. This hyperperfusion may represent a late response of cerebral autoregulation to increased ICP after saline-injection and the consequential shift of the subarachnoid milieu. If so, the absence of reactive hyperperfusion in the SAH group would be pathological and could either reflect persistent cerebral vasospasms, or disturbed cerebral autoregulation or both. These results are in line with the results from a previous study using the blood injection model, in which significant MCA constriction between 6 and 36 hours was reported [25]. However, occurrence of cerebral vasospasm seems to vary among the different models, as other investigators have described cerebral vasospasm until day 7 after injection [7], [26]. It is therefore possible, that the different SAH models elicit different time courses of the occurrence of cerebral vasospasms.

Furthermore, cerebellar perfusion was found to be significantly reduced 6 and 12 hours after SAH compared to both sham and saline-injection groups. Therefore, cerebellar hypoperfusion occurs in parallel to perfusion impairment in the cerebrum. However, cerebellar hypoperfusion may be promoted by the distribution of subarachnoid blood injected into the cisterna magna. The impact of hypoperfusion of the basilar supply area for human patients suffering from SAH must be investigated in further studies.

During DCI, we observed no significant differences in diameter of basal cerebral arteries in ink-perfused brains. In principle, cerebral vessels may be dilated by high pressure during perfusion, especially by viscous fluids. As we performed pre-experiments, kept the perfusion pressure constant at 70 mmHg and did not include gelatin in the perfusion mixture, we could exclude this possibility. More likely, impairment of cerebral perfusion during DCI could be caused by vasospasms of the microvasculature as opposed to narrowing of main basal cerebral arteries.

However, the present model gives additional insights into neuronal signaling after SAH: SAH animals displayed significantly increased absolute delta power i.e. ECoG slowing, 12 hours and 24 hours after SAH, which is also reflected by a reduction in ADR of SAH animals compared to saline-injected animals 24 hours after SAH, indicating ischemic injury. As rCBF is reduced in the SAH group at the same time and differences resolve after 72 hours parallel to normalized rCBF, slowing and decreased ADR most likely correlates to impaired cerebral perfusion. These results are in line with previous reports from patients. Recently, Stuart and colleagues reported a correlation between the extent of intracortical EEG-measured ADR decrease and the degree of cerebral vasospasms in poor-grade SAH patients [29]. Among several investigated EEG parameters for detection of cerebral hypoperfusion in poor-grade SAH patients, decreased ADR showed the strongest association with DCI [30]. These findings could also be confirmed in surface EEG recordings [31], [32]. At this point it is important to stress that this study found subarachnoid blood but not increased subarachnoid volume (saline group) to induce ECoG slowing, pointing toward direct influence of blood metabolites on neuronal signaling. Nevertheless, the role of pathologic electrical activity in SAH is part of an ongoing debate. Spreading depolarization is an early indicator of DCI after SAH and may itself promote cerebral hypoxia [33]–[35]. Spreading depolarization after SAH may promote (spreading) ischemia and spreading or non-spreading depression of brain electrical activity, which have been observed in both patients and animals models [34]–[37]. However, spreading depolarization can occur in SAH patients without angiographic vasospasms [38]. We observed periodically recurring short episodes of depression of activity within the 0.5–2 Hz range lasting 2–3 seconds in some SAH animals but not in saline-injected or sham-operated animals, likely reflecting spreading depolarization, which can be observed as abrupt-onset episodes of depression of electrical activity in the low-frequency range of the ECoG [15]. Although we only observed these phenomena in the SAH group, it is important to mention that two components of our anesthesia ketamine and midazolam have been shown to reduce and increase spreading depolarization respectively [39]. Nevertheless, this finding indicates that the present model allows investigation of spreading depolarization and its role in the pathomechanisms of DCI as well as pharmaceuticals influencing spreading depolarization.

Parallel to the rCBF reduction in the S1, sensory performance of mice in neurological tests was significantly impaired 12 and 24 hours after SAH, possibly directly linking rCBF reduction to corresponding deficits. Sensory performance of mice was impacted to a greater degree than motor performance after 12 and 24 hours. Neurological performance was not tested after 6 hours, as mice were still recovering from anesthesia and neurological impairment due cerebral vasospasms would not have been reliably distinguishable from narcosis effects. Interestingly, sensory deficits were only transient because after 72 hours sensory performance was no longer significantly different between all groups. However, SAH animals displayed significantly poorer total neurologic integrity compared to both control groups after 72 hours, indicating other persisting deficits. Also these results are in line with a previous report which described poorer neurological performance of SAH animals tested 72 hours after blood injection [7].

Finally, we acknowledge some limitations of the present study: Some parameters could not been assessed in the present study such as blood gases (in particular pCO_2_) and arterial blood pressure. Analysis of these parameters in addition to the performed evaluations would require additional equipment implanted into the mice and therefore additional surgeries e.g. craniotomies or surgeries to implant the transmitters. Subsequently, stress of the mice would be increased affecting the animal's health and quality of the data. Also, the size of mice might be a limitation. However, the impact of variables like pCO_2_ or arterial blood pressure must be evaluated in further studies. Furthermore, although bent ECoG electrode tips are fixed over the dural mater, micro lesions of underlying cortical tissue can occur due to pressure through the thin murine dural mater. However, neither neurological deficits nor ictal waveforms due to implantation of ECoG electrodes have been observed in past studies rendering this telemetric ECoG method popular and reliable [40]. The present analysis aimed to analyse the murine SAH injection model. However, a further head-to-head study comparing the endovascular puncture and the injection SAH model is still lacking and might be important, as specific method-immanent differences between both models have yet not been analyzed.

The present study underlines the great importance of performing both, sham and saline-injection control groups: Increasing volume in the subarachnoid space and thereby increased ICP or shifting the subarachnoid milieu appears to compromise cerebral perfusion. These physiological effects must be distinguished from effects evoked by subarachnoid blood and its metabolites. Furthermore, specific effects of the surrounding conditions like narcosis or the effect of surgery on physiological parameters and performance must be controlled using a sham group. Therefore, both control groups are indispensable. The possibility to perform both control groups represents a crucial advantage of the injection model, enabling distinction between the effect of blood metabolites and increased volume in the subarachnoid space which is not possible in the filament perforation model. Lastly, we were able to verify the accuracy of the cisterna magna injection and dispersion of blood in the subarachnoid space and entire ventricle system.

## Supporting Information

S1 Fig **Neurological examination after 12, 24 and 72 hours.** Mice in the SAH group displayed significant deficits in sensory tests compared to the saline injection group after 12 and 24 hours and compared to the sham-operated animals after 24 hours after SAH. After 3 days, significant sensory deficits were no longer visible in the SAH group. Motor deficits in the SAH group were significant compared to the sham-operated animals after 72 h.
(JPG)

S2 Fig **Relative rCBF impairment following acute SAH.** A: relative rCBF impairment in the somatosensory cortex following acute SAH. B: relative rCBF impairment in the cerebellar cortex following acute SAH. Blood injection caused instant severe hypoperfusion, whereas saline injection produced mild rCBF impairment in some animals or no impairment at all. No impairment was found in the sham group.
(JPG)

S3 Fig **India ink stained cerebral vessels.** A: Brain of a mouse transcardially perfused with india ink. B: Vessel diameter at 6 hours post injection. C: Vessel diameter at 24 hours post injection (Abbreviations: ACA: anterior cerebral artery, B: basilar artery, MCA: medial cerebral artery, PCA: posterior cerebral artery).
(JPG)

S1 Table **Scale used for neurological examination.** Neurologic deficits were examined using a scale previously described by Parra et al. (2002).
(XLSX)

S2 Table **Relative cerebral perfusion values derived from Laser Doppler measurements.** S2 Table summarizes the relative cerebral perfusion values related to the nadir to the pre-injection baseline, the nadir to the following peak and the preinjection to the postinjection baseline (Abbreviations: conc: Concentration of moving blood cells, n.d.: not determinated, rCBF: regional cerebral blood flow; SAH: subarachnoid hemorrhage, Saline: Saline-injection group, SEM: standard error of mean).
(XLSX)

S3 Table **Cerebral and cerebellar perfusion values during DCI.** S3 Table summarizes cerebral and cerebellar rCBF perfusion values 6, 12, 24 and 72 hour after ictus (Abbreviations: conc: Concentration of moving blood cells, d: day, rCBF: regional cerebral blood flow; SAH: subarachnoid hemorrhage, Saline: Saline-injection group, SEM: standard error of mean).
(XLSX)
