# Correction: Evaluation of a Murine Single-Blood-Injection SAH Model

**DOI:** 10.1371/journal.pone.0152244

**Published:** 2016-03-21

**Authors:** The PLOS ONE Editors

The article reports the recording of telemetric data from implanted TL11M2-F20-EET transmitters. These results are described in Figures 4 and 5.

The TL11M2-F20-EET transmitter has a reported transmission bandwidth of 1-50Hz and a sampling frequency of 250 Hz. The maximum identifiable signal for the device is 125 Hz.

The article reports experiments measuring signals of up to 500Hz. Given the limitations of the transmitter employed, the results reported for frequencies beyond 100Hz are not reliable. We are thus issuing this notification to alert readers that the results in Figure 4 and Figure 5C reporting signals over 100Hz are not reliable.
