# Supplementary figures and images for: Evaluation of a Murine Single-Blood-Injection SAH Model
Source: PLoS One. 2014 Dec 29;9(12):e114946. doi: 10.1371/journal.pone.0114946 (PMC4278886; doi:10.1371/journal.pone.0114946)

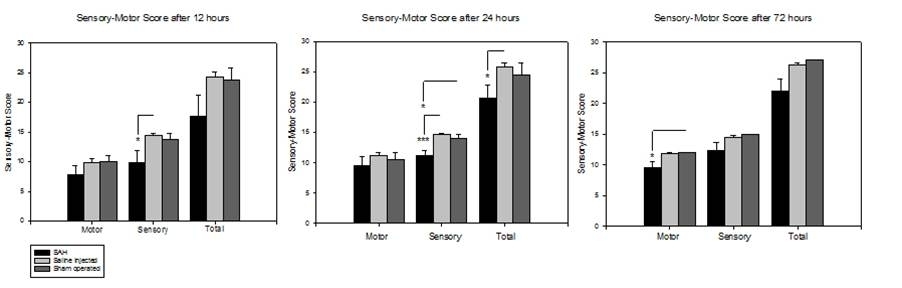

Supplement: S1 Fig — Neurological examination after 12, 24 and 72 hours. Mice in the SAH group displayed significant deficits in sensory tests compared to the saline injection group after 12 and 24 hours and compared to the sham-operated animals after 24 hours after SAH. After 3 days, significant sensory deficits were no longer visible in the SAH group. Motor deficits in the SAH group were significant compared to the sham-operated animals after 72 h. (JPG) [file pone.0114946.s001.jpg]

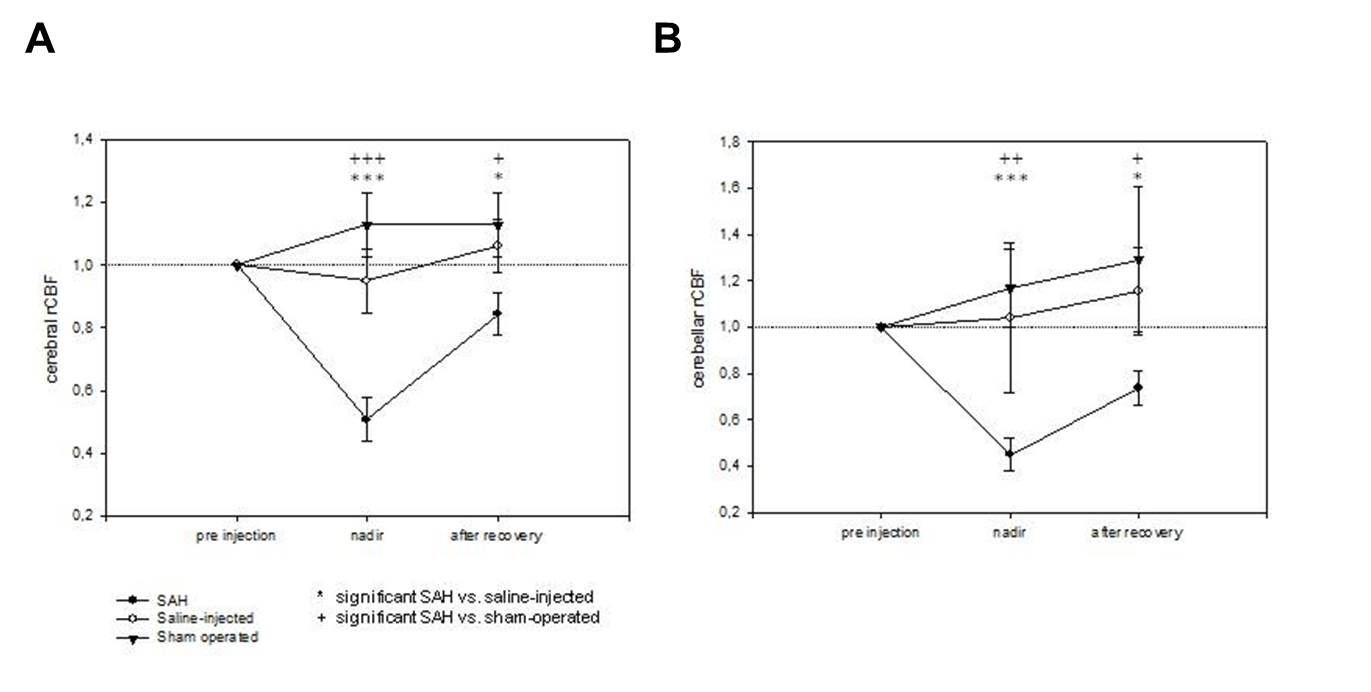

Supplement: S2 Fig — Relative rCBF impairment following acute SAH. A: relative rCBF impairment in the somatosensory cortex following acute SAH. B: relative rCBF impairment in the cerebellar cortex following acute SAH. Blood injection caused instant severe hypoperfusion, whereas saline injection produced mild rCBF impairment in some animals or no impairment at all. No impairment was found in the sham group. (JPG) [file pone.0114946.s002.jpg]

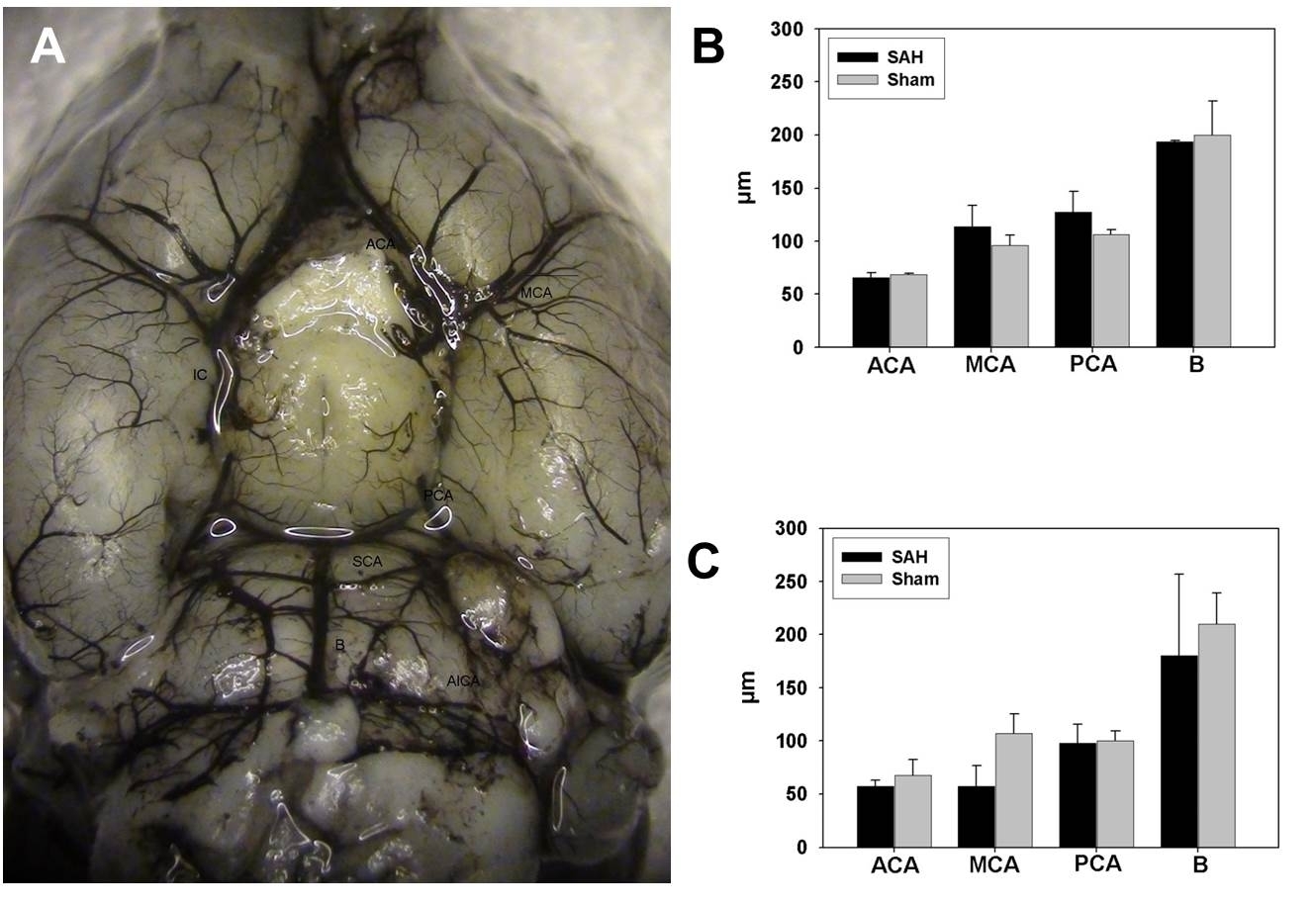

Supplement: S3 Fig — India ink stained cerebral vessels. A: Brain of a mouse transcardially perfused with india ink. B: Vessel diameter at 6 hours post injection. C: Vessel diameter at 24 hours post injection (Abbreviations: ACA: anterior cerebral artery, B: basilar artery, MCA: medial cerebral artery, PCA: posterior cerebral artery). (JPG) [file pone.0114946.s003.jpg]
